# Supplementary material for: Exploring the Role of BCL2 Interactome in Cancer: A Protein/Residue Interaction Network Analysis
Source: Biology (Basel). 2025 Mar 5;14(3):261. doi: 10.3390/biology14030261 (PMC11940271; doi:10.3390/biology14030261)
Supplement: Supplementary file 1 [file biology-14-00261-s001.zip › Supplementary MD figures and tables.pdf]

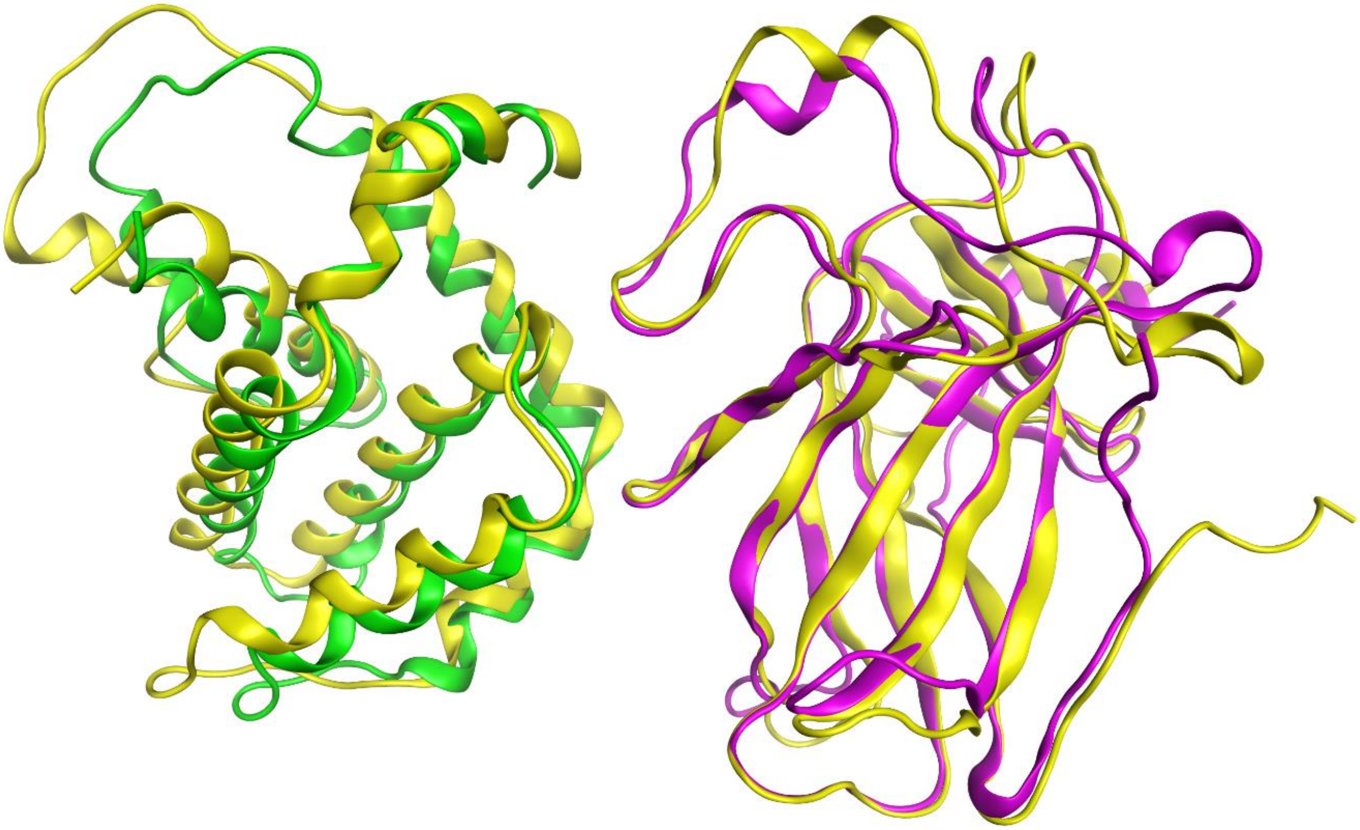

Supplementary Figure S1. Superimposed structures of BCL2-p53 complex (yellow) before and after 200 ns molecular dynamics (MD) simulations where green showed BCL2 protein and magenta color indicates p53.

Supplementary Table S11. Key residues involved in expanded contacts of BCL2-p53 identified before and after 200 ns MD simulations.

Docked complex				Molecular dynamics (MD) simulations			
**p53**	**BCL2**	**Energy (kcal/mol)**	**Area (Å²)**	**p53**	**BCL2**	**Energy (kcal/mol)**	**Area (Å²)**
Ser185	Tyr155	-0.01	7.14	Ser185	Leu154	-1.66	32.68
Asp186	Phe57	10.91	10.73	Asp186	Phe57	3.41	4.57
Asp186	Arg60	0.33	19.72	Asp186	Arg60	0.72	17.57
Asp186	Tyr155	-0.46	3.79	Asp186	Tyr61	0	6.78
Gly187	Asn96	0.05	0.99	Asp186	Gly98	-0.02	1.59
Gly187	Trp97	-0.07	0	Asp186	Leu154	-0.62	3.85
Gly187	Gly98	0.01	17.25	Asp186	Tyr155	-2.55	7.34
Gly187	Tyr155	152.34	19.23	Gly187	Asn96	0.08	10.3
Leu188	Tyr61	-0.75	14.6	Gly187	Trp97	0.48	9.75
Leu188	Asn96	-0.24	6.34	Gly187	Gly98	-1.26	8.66
Leu188	Gly98	-1.54	10.56	Gly187	Leu154	-1.6	18.15
Leu188	Arg99	-1.35	13.74	Gly187	Tyr155	0.18	3.21
Gly199	Arg60	-0.04	14.86	Leu188	Phe57	-0.72	11.35
Gly199	Tyr61	1.14	16.07	Leu188	Tyr61	-1.19	16.23
Gly199	Asp64	0.02	11.81	Leu188	Asn96	-0.56	24.75
Asn200	Tyr61	0.73	8.84	Leu188	Gly98	-1.4	11.53
Leu201	Tyr61	-3.25	30.3	Leu188	Arg99	-0.67	19.93
Leu201	Phe65	-1.13	13.03	Gly199	Arg60	-0.86	16.32
Leu201	Met68	-0.37	4.97	Gly199	Tyr61	0.03	10.07
Leu201	Leu90	-1.13	16.98	Gly199	Asp64	0.03	1.83
Leu201	Ala102	-1.7	15.65	Asn200	Tyr61	-0.12	11.23
Arg202	Glu67	-0.57	31.96	Asn200	Asp64	-0.21	6.64
Glu204	Asp93	60.45	52.75	Leu201	Tyr61	0.57	36.62
				Leu201	Asp64	-5.78	25.7
				Leu201	Phe65	-0.21	3.13
				Leu201	Met68	-0.71	11.32
				Leu201	Leu90	-1.41	18.13
				Leu201	Ala102	-1.15	16.32
				Arg202	Glu67	0	9.91
				Arg202	Met68	-1.01	15.52
				Arg202	Glu89	-0.46	14.15
				Arg202	Leu90	0.62	15.83
				Arg202	Arg92	0.41	16.08
				Val203	Arg99	-0.7	9.33
				Glu204	Asp93	-0.08	26.4

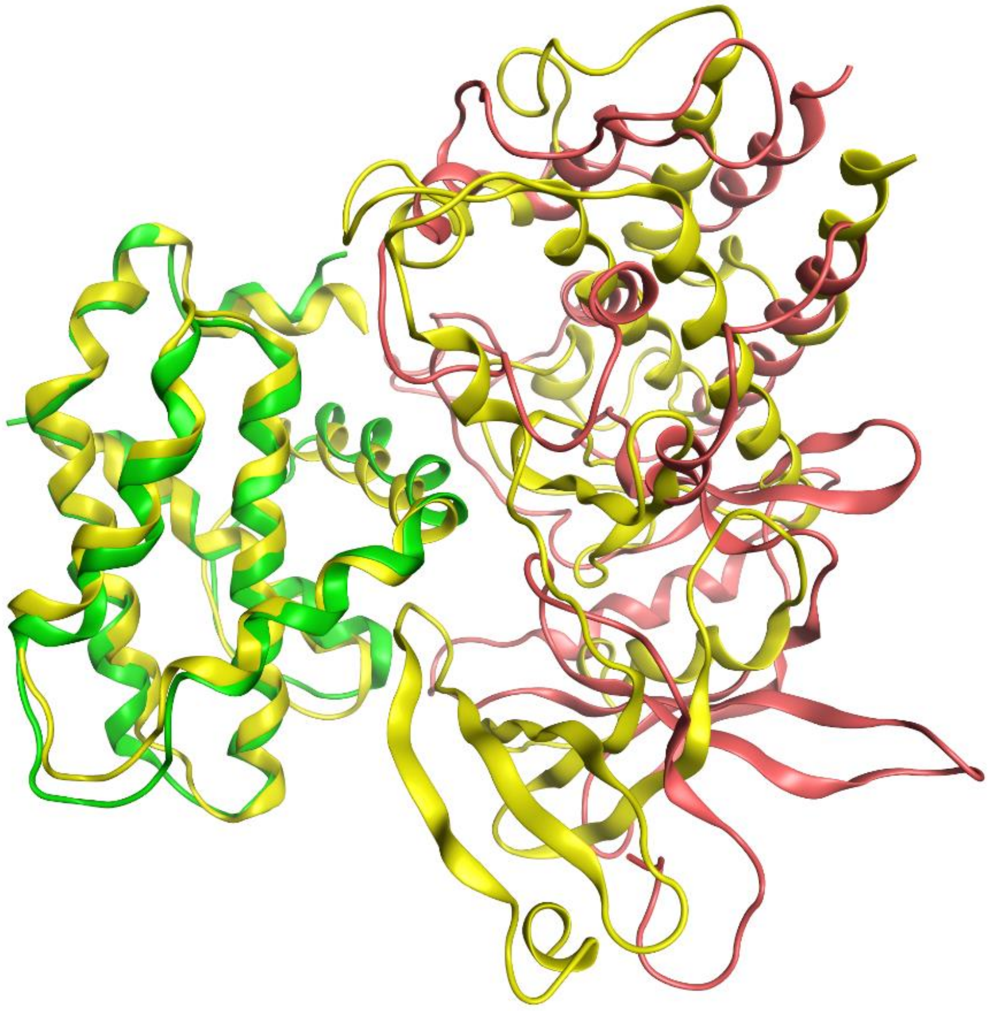

Supplementary Figure S2. Superimposed structures of BCL2-RAF1 complex (yellow) before and after 200 ns molecular dynamics (MD) simulations where green showed BCL2 protein and salmon indicates RAF1.

Supplementary Table S12. Key residues involved in expanded contacts of BCL2-RAF1 identified before and after 200 ns MD simulations.

Docked complex				Molecular dynamics (MD) simulations			
RAF1	BCL2	Energy (kcal/mol)	Area (Å²)	RAF1	BCL2	Energy (kcal/mol)	Area (Å²)
Arg354	Ala113	-0.76	15.7	Arg354	Ala113	-0.76	15.7
Arg354	Glu160	-0.17	14.33	Arg354	Glu160	-0.17	14.33
Ile355	Arg109	3.23	6.36	Ile355	Arg109	3.23	6.36
Ile355	Arg110	-1.91	34.77	Ile355	Arg110	-1.91	34.77
Ile355	Ala113	7.44	11.05	Ile355	Ala113	7.44	11.05
Gly356	Arg109	0.52	19.21	Gly356	Arg109	0.52	19.21
Gly356	Ala113	0.36	1.65	Gly356	Ala113	0.36	1.65
Ser357	Ser105	-0.94	13.15	Ser357	Ser105	-0.94	13.15
Ser357	Arg109	0.07	17.41	Ser357	Arg109	0.07	17.41
Ser357	Val156	1.36	25.82	Ser357	Val156	1.36	25.82
Gly358	Ser105	0.04	9.29	Gly358	Ser105	0.04	9.29
Gly358	Arg109	0.41	6.81	Gly358	Arg109	0.41	6.81
Ser359	Lys22	0.1	3.76	Ser359	Lys22	0.1	3.76
Ser359	Gln25	-0.9	27.05	Ser359	Gln25	-0.9	27.05
Ser359	Asp102	-0.13	5.55	Ser359	Asp102	-0.13	5.55
Ser359	Ser105	-0.01	6.44	Ser359	Ser105	-0.01	6.44
Gly361	Arg26	0.14	8.81	Gly361	Arg26	0.14	8.81
Gly361	Arg109	-5.4	10.79	Gly361	Arg109	-5.4	10.79
Thr362	Arg109	0.02	1.38	Thr362	Arg109	0.02	1.38
Val363	Arg109	30.67	24.31	Val363	Arg109	30.67	24.31
Asp381	Arg26	-0.05	15.64	Asp381	Arg26	-0.05	15.64
Cys424	Arg110	0	8.42	Cys424	Arg110	0	8.42
Glu425	Arg110	-0.11	17.06	Glu425	Arg110	-0.11	17.06
Ser427	Arg110	-0.2	30.42	Ser427	Arg110	-0.2	30.42
Ser428	Arg107	-1.94	14.45	Ser428	Arg107	-1.94	14.45
Tyr430	Phe104	-0.14	9.24	Tyr430	Phe104	-0.14	9.24
Tyr430	Arg107	12.22	33.59	Tyr430	Arg107	12.22	33.59
Tyr430	Tyr108	43.69	24.25	Tyr430	Tyr108	43.69	24.25
Lys431	Arg107	-2.67	14	Lys431	Arg107	-2.67	14
Lys431	Tyr108	-0.65	12.15	Lys431	Tyr108	-0.65	12.15
Lys431	Arg109	0.1	0	Lys431	Arg109	0.1	0
Lys431	Arg110	0.65	10.17	Lys431	Arg110	0.65	10.17
Lys431	Asp111	29.23	16.32	Lys431	Asp111	29.23	16.32
His434	Tyr108	0	5.1	His434	Tyr108	0	5.1
His434	Arg146	0.55	31.35	His434	Arg146	0.55	31.35
Gln436	Asp140	0	20.3	Gln436	Asp140	0	20.3
Gln436	Arg146	0.06	11.75	Gln436	Arg146	0.06	11.75

Asp468	Leu106	-0.08	4.77	Asp468	Leu106	-0.08	4.77
Lys470	Asp103	-11.84	19.7	Lys470	Asp103	-11.84	19.7
Lys470	Leu106	-0.16	13.06	Lys470	Leu106	-0.16	13.06
Asn472	Asp103	-0.22	9.56	Asn472	Asp103	-0.22	9.56
Asn472	Leu106	0.01	11.77	Asn472	Leu106	0.01	11.77
Asn472	Arg107	33788.97	37.51	Asn472	Arg107	33788.97	37.51
Asn473	Leu106	-0.44	20.83	Asn473	Leu106	-0.44	20.83
Asp486	Leu106	-0.36	20.27	Asp486	Leu106	-0.36	20.27
Ser508	Gln99	-0.12	20.99	Ser508	Gln99	-0.12	20.99
Val509	Gln99	26.35	25.93	Val509	Gln99	26.35	25.93
Val509	Asp103	-0.13	2.61	Val509	Asp103	-0.13	2.61
Val509	Tyr202	421.73	16.03	Val509	Tyr202	421.73	16.03
Leu510	Tyr202	0.18	9.71	Leu510	Tyr202	0.18	9.71
Trp511	Asp103	-0.85	13.96	Trp511	Asp103	-0.85	13.96
Trp511	Arg107	131.73	22.6	Trp511	Arg107	131.73	22.6
Tyr548	Trp144	-0.08	28.31	Tyr548	Trp144	-0.08	28.31
Tyr548	Leu201	379.97	26.16	Tyr548	Leu201	379.97	26.16
Arg554	Asn143	0.34	12.65	Arg554	Asn143	0.34	12.65
Arg554	Gly145	0.2	12.42	Arg554	Gly145	0.2	12.42
Asp555	Leu201	-1.32	33.41	Asp555	Leu201	-1.32	33.41
Phe559	Glu200	-0.1	6.89	Phe559	Glu200	-0.1	6.89
Phe559	Leu201	0.18	22	Phe559	Leu201	0.18	22

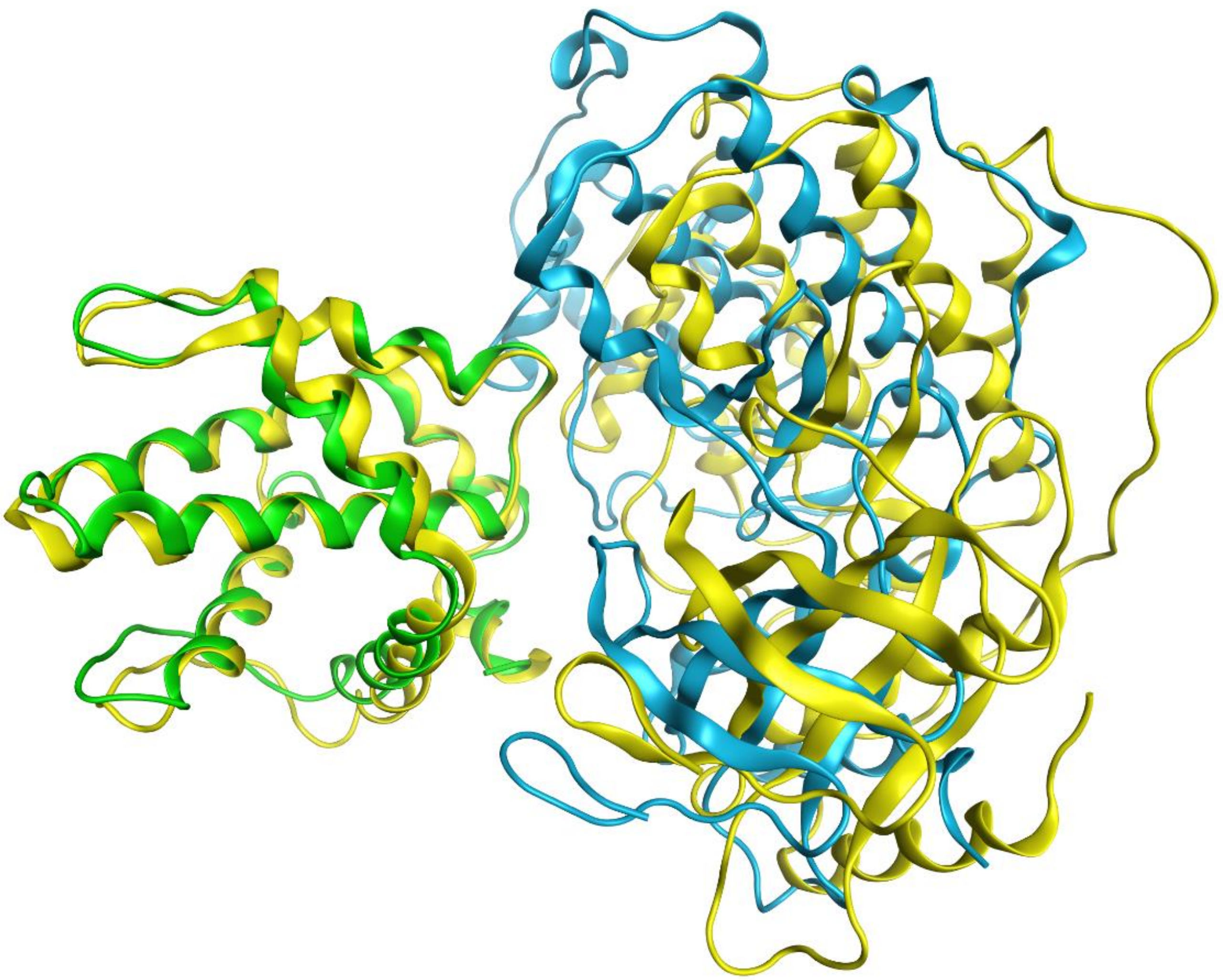

Supplementary Figure S3. Superimposed structures of BCL2-MAPK1 complex (yellow) before and after 200 ns molecular dynamics (MD) simulations where green showed BCL2 protein and cyan color indicates MAPK1.

Supplementary Table S13. Key residues involved in expanded contacts of BCL2-MAPK1 identified before and after 200 ns MD simulations.

Docked complex				Molecular dynamics (MD) simulations			
MAPK1	BCL2	Energy (kcal/mol)	Area (Å^2)	MAPK1	BCL2	Energy (kcal/mol)	Area (Å^2)
Arg13	Asp40	0.1	11.53	Arg13	Asp40	0.1	11.53
Arg13	Leu43	8.37	10.42	Arg13	Leu43	8.37	10.42
Arg13	Arg44	22303.48	62.82	Arg13	Arg44	22303.48	62.82
Arg13	Tyr45	0.19	0	Arg13	Tyr45	0.19	0
Tyr28	Arg44	5299.37	42.2	Tyr28	Arg44	5299.37	42.2
Tyr28	Tyr45	-0.52	7.4	Tyr28	Tyr45	-0.52	7.4
Ile29	Tyr45	0	4.55	Ile29	Tyr45	0	4.55
Glu31	Phe41	-0.19	6.87	Glu31	Phe41	-0.19	6.87
Glu31	Tyr45	3.69	23.11	Glu31	Tyr45	3.69	23.11
Met36	Arg44	201.94	19.81	Met36	Arg44	201.94	19.81
His59	Glu137	-0.71	43.99	His59	Glu137	-0.71	43.99
Tyr62	Leu138	46.72	46.16	Tyr62	Leu138	46.72	46.16
Tyr111	Glu73	0	1.24	Tyr111	Glu73	0	1.24
Tyr111	Leu74	1.08	3.91	Tyr111	Leu74	1.08	3.91
Tyr111	Phe75	-0.15	1.41	Tyr111	Phe75	-0.15	1.41
Tyr111	Arg76	-0.25	3.37	Tyr111	Arg76	-0.25	3.37
Tyr111	Asp77	22605.71	47.26	Tyr111	Asp77	22605.71	47.26
Tyr111	Gly78	0.2	0	Tyr111	Gly78	0.2	0
Tyr111	Arg83	21469.67	36.1	Tyr111	Arg83	21469.67	36.1
Leu114	Arg76	0	8.31	Leu114	Arg76	0	8.31
Lys115	Glu73	2.95	52.82	Lys115	Glu73	2.95	52.82
Lys115	Arg76	0.28	20.72	Lys115	Arg76	0.28	20.72
Lys115	Asp77	-0.75	5.95	Lys115	Asp77	-0.75	5.95
Pro150	Asp77	0.24	5.91	Pro150	Asp77	0.24	5.91
Ser151	Asp77	0	5	Ser151	Asp77	0	5
Glu184	Asn129	-0.21	1.56	Glu184	Asn129	-0.21	1.56
Tyr185	Asn80	-0.59	5.9	Tyr185	Asn80	-0.59	5.9
Tyr185	Trp81	29.9	24.71	Tyr185	Trp81	29.9	24.71
Tyr185	Trp125	257.51	16.61	Tyr185	Trp125	257.51	16.61
Tyr185	Asn129	1285.54	25.61	Tyr185	Asn129	1285.54	25.61
Tyr185	Leu138	-0.24	10.15	Tyr185	Leu138	-0.24	10.15
Val186	Val79	0.02	0	Val186	Val79	0.02	0
Val186	Trp125	22.53	11.99	Val186	Trp125	22.53	11.99
Val186	Asn129	31.23	10.29	Val186	Asn129	31.23	10.29
Ala187	Gly78	-0.23	1.11	Ala187	Gly78	-0.23	1.11
Ala187	Val79	510686.7	10.26	Ala187	Val79	510686.7	10.26
Ala187	Asn80	144.42	35.31	Ala187	Asn80	144.42	35.31

Ala187	Trp125	-0.9	3.61	Ala187	Trp125	-0.9	3.61
Thr188	Asp77	-0.1	1.36	Thr188	Asp77	-0.1	1.36
Thr188	Gly78	6.33	19.76	Thr188	Gly78	6.33	19.76
Thr188	Val79	-0.27	2.69	Thr188	Val79	-0.27	2.69
Thr188	Trp125	0.19	3.09	Thr188	Trp125	0.19	3.09
Arg189	Trp125	0.81	14.75	Arg189	Trp125	0.81	14.75
Arg189	Asp128	-0.01	0.62	Arg189	Asp128	-0.01	0.62
Arg189	Asn129	-0.99	12.58	Arg189	Asn129	-0.99	12.58
Trp190	Phe75	-0.42	3.27	Trp190	Phe75	-0.42	3.27
Trp190	Arg76	170.19	14.7	Trp190	Arg76	170.19	14.7
Trp190	Asp77	-0.53	0	Trp190	Asp77	-0.53	0
Trp190	Gly78	-0.41	12.37	Trp190	Gly78	-0.41	12.37
Trp190	Val79	-0.71	12.09	Trp190	Val79	-0.71	12.09
Tyr191	Asp77	-0.09	9.59	Tyr191	Asp77	-0.09	9.59
Glu218	Arg76	0	1.25	Glu218	Arg76	0	1.25
Glu218	Asp77	0	4.15	Glu218	Asp77	0	4.15
Asn222	Arg76	-0.1	20.05	Asn222	Arg76	-0.1	20.05
Pro224	Arg76	0.28	15.68	Pro224	Arg76	0.28	15.68
Lys229	Asp120	-1.68	21.7	Lys229	Asp120	-1.68	21.7
Lys229	His121	0.08	21.5	Lys229	His121	0.08	21.5
Lys229	Thr124	4209.34	29.83	Lys229	Thr124	4209.34	29.83
Lys229	Trp125	-0.37	5.68	Lys229	Trp125	-0.37	5.68
Lys229	Asp128	0.22	2.43	Lys229	Asp128	0.22	2.43
His230	Thr124	662.64	42.76	His230	Thr124	662.64	42.76
His230	Asp128	10.62	18.57	His230	Asp128	10.62	18.57
Tyr231	Asp128	-1.35	36.14	Tyr231	Asp128	-1.35	36.14
